# Reproductive hormones mediate changes in the gut microbiome during pregnancy and lactation in Phayre’s leaf monkeys

**DOI:** 10.1038/s41598-020-66865-2

**Published:** 2020-06-19

**Authors:** Elizabeth K. Mallott, Carola Borries, Andreas Koenig, Katherine R. Amato, Amy Lu

**Affiliations:** 1 Department of Anthropology, Northwestern University, Evanston, IL USA; 2 Department of Anthropology, Stony Brook University, Stony Brook, NY USA; 3 Interdepartmental Doctoral Program in Anthropological Sciences, Stony Brook University, Stony Brook, NY USA

**Keywords:** Microbial communities, Biological anthropology, Reproductive biology, Microbial ecology

## Abstract

Studies in multiple host species have shown that gut microbial diversity and composition change during pregnancy and lactation. However, the specific mechanisms underlying these shifts are not well understood. Here, we use longitudinal data from wild Phayre’s leaf monkeys to test the hypothesis that fluctuations in reproductive hormone concentrations contribute to gut microbial shifts during pregnancy. We described the microbial taxonomic composition of 91 fecal samples from 15 females (n = 16 cycling, n = 36 pregnant, n = 39 lactating) using 16S rRNA gene amplicon sequencing and assessed whether the resulting data were better explained by overall reproductive stage or by fecal estrogen (fE) and progesterone (fP) concentrations. Our results indicate that while overall reproductive stage affected gut microbiome composition, the observed patterns were driven by reproductive hormones. Females had lower gut microbial diversity during pregnancy and fP concentrations were negatively correlated with diversity. Additionally, fP concentrations predicted both unweighted and weighted UniFrac distances, while reproductive state only predicted unweighted UniFrac distances. Seasonality (rainfall and periods of phytoprogestin consumption) additionally influenced gut microbial diversity and composition. Our results indicate that reproductive hormones, specifically progestagens, contribute to the shifts in the gut microbiome during pregnancy and lactation.

## Introduction

The gut microbiome interacts closely with host physiological processes, including growth, metabolism, and immunity^1^. Because many of these processes dynamically change in response to reproduction, alterations in the female gut microbiome are expected across reproductive stages. In support of this expectation, shifts in gut microbial diversity and composition during pregnancy and lactation have been reported in a number of vertebrates^2–5^, including humans^6–8^ and nonhuman primates^9,10^. However, not all studies of humans have consistently found changes in the gut microbiome during pregnancy and lactation^11,12^. Additionally, the direction of change and the specific taxa involved vary across studies. For instance, in bats, gut bacterial diversity was significantly higher in pregnant and lactating females^2^, while in captive mice and oviparous lizards, pregnancy reduced alpha diversity^3,4^. Similarly, in humans, one study reported decreases in diversity over the course of pregnancy^8^, and two others reported no change in diversity between the third trimester and postpartum^6,13^.

Little is known about the proximate mechanisms underlying shifts in the gut microbiome across reproductive state. While potential drivers include diet, behavior, and social factors, several authors have suggested that reproductive hormones are likely mediating these changes^6,9,14^. Reproductive hormones - including progestagens and estrogens - orchestrate a number of physiological changes during pregnancy, including alterations in inflammatory immune responses, as well as changes in maternal metabolism and cardiovascular function^15^. These hormones are therefore likely candidates for mediating changes in the gut microbiome. Limited evidence suggests that estrogens, in particular, can drive shifts in gut microbial composition^16,17^. However, few studies have simultaneously measured reproductive hormone concentrations and gut microbial community composition across cycling, pregnant, and lactating individuals^5^.

Knowledge of these interactions is critical for our understanding of host physiology, health, ecology and evolution. Mammalian female reproduction is strongly constrained by energy, and females are known to employ varied behavioral (e.g., increasing intake, decreasing activity) and physiological strategies (e.g., allocating energy away from immunity) to decrease daily energy consumption of non-reproductive functions and increase the energy available for reproduction^18–21^. Thus, changes in the gut microbiome during pregnancy and lactation may have a similar function: commensal relationships with gut microbiota may help females compensate for energetic shortfalls during reproduction^1,9,22^. Nevertheless, how shifts in the gut microbiome during reproduction alter the energy available to the host is not well defined. To our knowledge, only a single paper provides direct evidence of this with some features of the gut microbiome during the third trimester of pregnancy resembling features of the obesity-associated gut microbiome; however, the amount of energy lost in the stool increases during the third trimester, indicating that the gut microbiome may be increasing energy harvest but not the efficiency of energy uptake^8^. Alternatively, microbial shifts may result from other host environmental or physiological changes during pregnancy and lactation which may not directly relate to energy metabolism^22^. Additional research is necessary to distinguish these scenarios.

To begin to address these gaps, this study uses a wild folivorous primate - the Phayre’s leaf monkey (*Trachypithecus phayrei crepusculus*) - as a model to examine relationships between adult female reproductive state, reproductive hormones, and the gut microbiome. If shifts in the gut microbiome support the metabolic requirements of pregnancy and lactation, they are likely to be more marked in wild populations where individuals are energetically constrained, making them easier to detect. Additionally, because leaf monkeys are folivores with low-energy diets and a gut physiology that precludes consumption of large amounts of ripe, sugar-rich fruit^23^, they are expected to face exaggerated energetic challenges during costly reproductive states compared to other primates. Building on existing research on the reproductive endocrinology of this population, we examined how the composition of the gut microbiome varied across reproductive stages and tested whether observed patterns were associated with differences in fecal estrogen (fE) and progestagen (fP) concentrations. Specifically, we hypothesized that variations in reproductive hormone concentrations would better predict shifts in the gut microbiome than coarse categories of reproductive state (cycling, pregnant, lactating) in adult female Phayre’s leaf monkeys.

## Methods

### Study site

**Figure 1:**
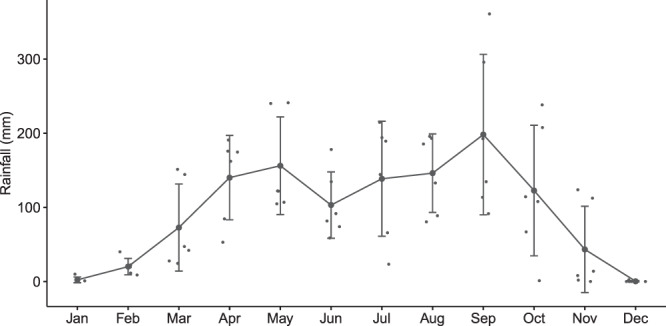
Average monthly rainfall from 2003–2008 at the study site. Confidence intervals show one standard deviation from the mean, and the jitter points show individual data points.

Data for this study were collected as part of a long-term project (directed by A. Koenig and C. Borries) on the behavioral ecology of wild Phayre’s leaf monkeys in Phu Khieo Wildlife Sanctuary, Thailand (16°5′-35′N, 101°20′-55′E, Chaiyaphum Province, elevation: 300–1300 m above sea level). Annual rainfall averaged 1144 mm^24^, with most rain falling during a wet season from April through October with March and November as transitional months (Fig. 1). Although births occurred in all months of the year in this population, most births were concentrated in the period between November and April (Borries *et al*., unpublished).

### Sample collection

Fecal samples were collected as part of a study on reproductive physiology from two groups (PA and PB) between February 2005 - September 2006. All samples were stored in a cooler while in the field, and transferred to a −20 °C freezer upon return to the field site. All frozen samples were then transferred to the US, where they were freeze-dried and sifted into fine powder at the Center for Conservation and Reproduction of Endangered Species in San Diego. Of the original 2000 collected fecal samples, we analyzed 91 samples from 15 females (PA: n = 5 females, n = 24 fecal samples; PB: n = 10 females, n = 67 fecal samples) to determine gut microbiome composition. For each female, we selected multiple representative samples from each of the 3 reproductive periods — cycling, pregnancy, and lactation — available for that female (mean = 5.47 ± 2.07 samples per individual). Selected samples were distributed evenly within a reproductive period (e.g., one sample was taken from each of the 1st, 2nd, and 3rd trimesters of pregnancy). A subset of 68 gut microbiome samples from 10 individuals were matched with samples analyzed for hormone concentrations to assess the relationships between fE and fP and the gut microbiome (see below). Cycling, pregnancy, and lactation were defined from hormone patterns available for 10 individuals^25^. For the remaining 3 individuals, the beginnings of pregnancy and lactation were defined as the date of conception and the date of parturition, respectively, where the former was estimated by subtracting the mean gestation length for the population (205.3 days)^25^ from the exact date of birth. Although a population average of 3.57 cycles to conception^25^ suggests that these 3 females could have been cycling for 100 days prior to the calculated conception date, we only used samples from the two weeks prior to conception to assure that we were sampling from physiologically cycling females.

### DNA extraction

Lyophilized samples were extracted with a DNAeasy PowerSoil Kit (Qiagen) following the manufacturer’s protocol, with the following modifications: (1) The input sample weight was reduced to 0.1 g to account for the dehydrated nature of the samples; (2) After the addition of solution C1, samples were incubated at 65 °C for 20 minutes; and (3) Solution C6 was warmed to 65 °C prior to adding it to the spin columns, followed by a 5-minute incubation at room temperature prior to centrifugation.

### 16S rRNA gene amplification and sequencing

The V4-V5 region of the 16S rRNA gene was amplified using the 515Fa-926R Earth Microbiome Project primer set^26,27^ in a two-step PCR reaction following published lab protocols^9^. Both extraction and PCR negatives were performed to control for contamination. SequelPrep was used to purify and normalize PCR products. Samples were sequenced on the Illumina MiSeq V3 platform (2×300 bp) at the University of Illinois Chicago’s Sequencing Core.

### Sequence processing and analysis

Sequencing yielded 3,205,004 total reads with an average of 33,385.46 reads per sample (range = 2,467–46,337). Sequences were trimmed, quality-filtered, and dereplicated; amplicon sequence variants (ASVs) were inferred; and paired reads were merged using the DADA2 algorithm^28^ within QIIME2 (v2019.4)^29^. Details of the quality-filtering and trimming parameters can be found in the supplementary material. Taxonomy was assigned in QIIME2 using a Naive Bayes classifier trained on the Greengenes 13_8 99% OTU database using the full 16S rRNA gene sequence lengths. Mitochondria and chloroplast ASVs were filtered from the dataset, and two samples that did not cluster with the others were removed. After all filtering steps, our dataset contained 1,023,106 total ASVs (2,669 unique ASVs) with an average of 11,242.92 ASVs per sample (range 1,003–17,777).

Alpha diversity and beta diversity were calculated in QIIME2. For alpha diversity, we used both observed OTUs as a measure of estimated richness and the Shannon diversity index to account for both the abundance and evenness of ASVs. In order to incorporate phylogenetic information, unweighted (presence/absence) and weighted (corrected for abundance) UniFrac distances were used for beta diversity calculations. Samples were rarefied to 8,000 reads per sample prior to alpha and beta diversity calculations to allow for adequate sequencing depth while still maintaining even sampling across treatment groups, resulting in nine samples being excluded from the dataset. We present analyses of beta diversity metrics calculated from relative abundances here. Beta diversity analyses of unrarefied data and DESeq.2^30^-normalized abundances for individual taxa can be found in the supplementary material.

We explored the use of PICRUSt2 to predict MetaCyc reaction pathway abundances from 16S rRNA gene sequences^31–35^. However, after dropping sequences with a NSTI score >2, the average weighted NSTI score (a measure of the branch length distance between the observed sequence and the closest sequence in the reference database) was 0.244 ± 0.025, a higher weighted NSTI score than what is deemed acceptable (range <0.15) for functional inferences. Furthermore, 88% of sequences could not be assigned to genus. We therefore opted to not infer function from incomplete and poorly mapped data.

### Hormone analysis

Fecal progestin (fP) and estrogen (fE) metabolites were extracted and analyzed via radioimmunoassay (n = 68) as previously described^25,36^. As previously reported based on a larger sample size (n = 2077), female Phayre’s leaf monkeys were consuming plants (*Vitex* spp.) containing phytoprogestins from May-Sept 2005 and April-Sept 2006, increasing their excreted fP metabolite concentrations during these periods^36^. Although it is unknown whether measured fP concentrations during this time reflect phytoprogestin concentrations in the plant itself (measured in the fiber content of the feces) or phytoprogestins assimilated into circulation, the reproductive function of cycling females (cycle length and conception probability) was altered during this period, suggesting that at least some of the plant hormones influenced endogenous hormone concentrations. To be cautious, we control for phytoprogestin consumption in all analyses examining the effect of fP on gut microbiome concentrations.

Measurements of fecal hormone metabolites represent circulating hormone concentrations from 24 hours prior to fecal sample collection within a single individual, though other studies suggest a more conservative estimate of 24–48 hours to account for between-individual variation^25^. Additionally, hormone concentrations are highly variable and it is likely the average effect over time that influences host physiology and, presumably, the gut microbiome on a broad scale. To conservatively control for lag and to account for day-to-day variability, hormone concentrations were averaged across a 7-day period (date of sample collection ±3 days) whose midpoint date was determined by the date of the matched fecal sample for which microbiome data were available. Average 7-day hormone concentrations were then log-transformed for analyses.

### Statistical analysis

Linear mixed effects models were used to test for the effect of reproductive state and hormone concentrations on alpha diversity (*nlme* package, R)^37,38^. All models included reproductive state and rainfall as fixed effects and individual ID as a random effect to control for repeated measures. Rainfall was included as a fixed effect to control for seasonality. Models were additionally run with and without fP and fE as fixed effects in order to determine whether reproductive state had an effect independent of the effect of hormonal changes. For models with fP and fE, we also included the interaction between phytoprogestin period and fP to test if phytoprogestins were influencing the interactions we saw between fP and alpha diversity. Marginal and conditional R^2^ values were calculated to assess goodness of fit for linear mixed effects models (*performance* and *lme4* packages, R)^39,40^. Permutational Analysis of Variance tests (PERMANOVAs) were used to examine the influence of reproductive state and hormone levels on beta diversity (*vegan* package, R)^41^. As in the above, all models included reproductive state and rainfall as factors and individual ID as a “strata” to control for repeated measures. Models were also run with and without fP and fE as fixed effects, again including the interaction between fP and phytoprogestin period in the models with reproductive hormones. 5000 permutations were used in the analysis. Finally, pairwise PERMANOVAs were used to examine pairwise differences in beta diversity between specific reproductive states (*pairwiseAdonis* package, R)^42^.

Generalized linear mixed effects models using a negative binomial distribution were used to test for the effect of reproductive state and hormone concentrations on the relative abundance of individual taxa (*glmmTMB* and *car* packages, R)^43,44^. To account for low abundance samples, generalized linear mixed effects models using a binomial distribution were used to examine how reproductive state and hormone concentrations influenced the presence or absence of individual taxa (*lme4* package, R)^45^. While zero-inflated negative binomial and beta regression models are more appropriate for microbiome data, our small sample size limited the number of models using those distributions that converged. All models included reproductive state and rainfall (millimeters per month) as fixed effects and individual ID as a random effect to control for repeated measures. In addition, the models were run with and without hormonal covariates–fP, the interaction between fP and phytoprogestin period, and fE–as fixed effects. The results of family-level and genus-level models were corrected for false discovery rate (FDR) (*fdrtool* package, R)^46^. Pairwise comparisons between reproductive states were performed using Tukey contrasts with p-value adjustment based on the joint normal distribution and simultaneous confidence interval estimates (*multcomp* package, R)^47^. All statistical tests were performed in R (v3.6.0)^38^. Rainfall, reproductive state, and hormone data for the analysis are available in Dryad^48^.

### Ethics statement

All methods were carried out in accordance with relevant guidelines and regulations. Methods and protocols were approved by the Stony Brook University IACUC (IDs: 20041120 to 20061120) and complied with the laws of Thailand and the USA. The National Research Council of Thailand, the Department of National Parks, Wildlife, and Plant Conservation, and Phu Khieo Wildlife Sanctuary gave permission to conduct research in Thailand.

## Results

### Microbial diversity

**Figure 2:**
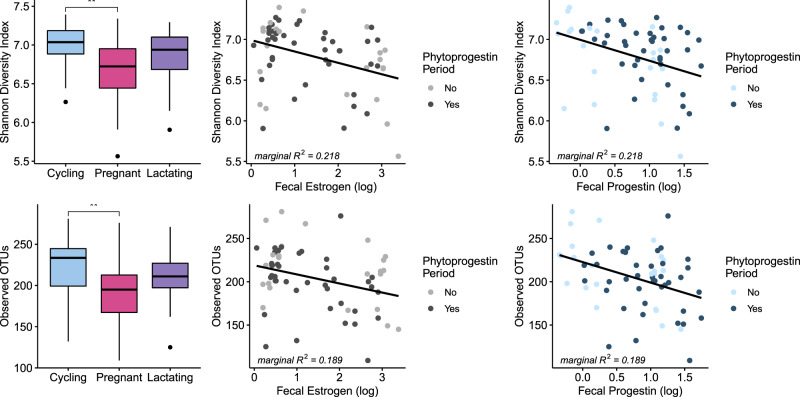
Relationship between reproductive state, fE concentrations, fP concentrations, and phytoprogestin period and Shannon Diversity index and observed OTUs. In the box plots line = median, box = interquartile range (IQR), whiskers = 1.5xIQR, and points denote outliers beyond the 1.5xIQR limit. In the scatterplots, marginal R^2^ values are reported for the full model.

In models including only reproductive state and rainfall (season), rainfall did not have a significant effect on alpha diversity (all p > 0.05) (Supplementary Table S1), but reproductive state had a significant influence on Shannon diversity index (Χ^2^ = 7.603, p = 0.022) and observed OTUs (Χ^2^ = 7.932, p = 0.019, Fig. 2). Pairwise comparisons showed that pregnant individuals had significantly lower Shannon diversity values and observed OTUs compared with cycling individuals (z = −2.569, p = 0.027 and z = −2.644, p = 0.022).

In models that also included hormone concentrations, rainfall, reproductive status and fE concentrations did not significantly influenced alpha diversity and fP concentration did not predict numbers of observed OTUs (all p > 0.05), but fP concentrations did negatively predicted Shannon diversity index values (Χ^2^ = 4.311, p = 0.038, Fig. 2, Supplementary Table S1). Periods of phytoprogestin consumption were not associated with Shannon diversity or observed OTUs (both p > 0.05, Supplementary Table S1), and there was no interaction effect between phytoprogestin period and fP concentrations on either measure of alpha diversity (Shannon: Χ^2^ = 0.035, p = 0.852; observed OTUs: Χ^2^ = 0.020, p = 0.889, Supplementary Table S1, Fig. 2).

### Microbial composition

**Figure 3:**
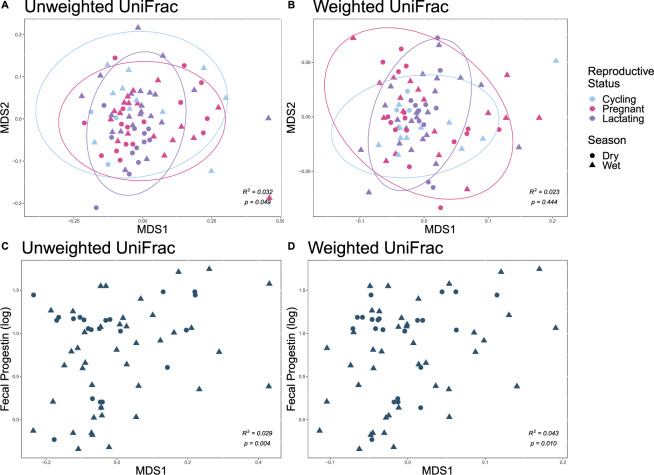
Nonlinear multidimensional scaling plot of unweighted and weighted UniFrac distances showing differences between reproductive states (**A**,**B**) and plotted against fP concentration (**C**,**D**).

For models without hormone concentrations, rainfall had a significant effect on unweighted and weighted UniFrac distances (unweighted: F_1,78_ = 2.681, R^2^ = 0.032, p < 0.001; weighted: F_1,78_ = 5.172, R^2^ = 0.061, p = 0.001, Supplementary Table S2, Fig. 3). Reproductive status had a significant effect on unweighted UniFrac distances (F_2,78_ = 1.313, R^2^ = 0.032, p = 0.049), but not on weighted UniFrac distances (p > 0.05, Supplementary Table S2, Fig. 3). Pairwise comparisons between each pair of reproductive states were not significant for either unweighted or weighted UniFrac distances after adjusting for multiple comparisons.

When we included hormone concentrations in the model, rainfall still had a significant effect on unweighted UniFrac distances (F_1,55_ = 1.482, R^2^ = 0.022, p = 0.027), but not weighted UniFrac distances (F_1,55_ = 1.704, R^2^ = 0.025, p = 0.062, Supplementary Table S2, Fig. 2). Reproductive status did not have a significant influence on unweighted or weighted UniFrac distances (all p > 0.05, Supplementary Table S2, Fig. 3). fP significantly influenced both unweighted and weighted Unifrac distances (F_1,55_ = 1.927, R^2^ = 0.029, p = 0.004 and F_1,55_ = 2.983, R^2^ = 0.043, p = 0.010), but fE did not (all p > 0.05, Supplementary Table S2, Fig. 3). Although the phytoprogestin period had a significant influence on both unweighted and weighted UniFrac distances (F_1,55_ = 2.980, R^2^ = 0.045, p < 0.001 and F_2,55_ = 5.317, R^2^ = 0.077, p < 0.001), the interaction between fP and phytoprogestin period was not significant for either measure (all p > 0.05, Supplementary Table S2, Fig. 3).

### Taxonomic composition

The relative abundances of bacterial phyla were not significantly influenced by rainfall, reproductive state, fP, fE, or phytoprogestin period in any model (all p > 0.05). At the family level, rainfall was positively correlated with the relative abundance of Peptococcaceae (Firmicutes) (Χ^2^ = 24.767, p < 0.001, q < 0.001), and, at the genus level, rainfall was positively correlated with the relative abundance of *Eubacterium* (Firmicutes) (Χ^2^ = 4.387, p = 0.036, q > 0.05), the rc4.4 genus within Peptococcaceae (Firmicutes) (Χ^2^ = 6.974, p = 0.008, q > 0.05), and *Coprobacillus* (Firmicutes) (Χ^2^ = 12.922, p < 0.001, q > 0.05), but only in models excluding hormones. The relative abundance of any bacterial families or genera were not significantly influenced by reproductive state, fP, fE, or phytoprogestin period prior to or after FDR correction in any model (all q > 0.05).

Binomial models used to test whether any variables predicted the presence or absence of taxa showed that reproductive status, fP, and fE did not predict the occurrence of any phyla, families, or genera in the gut microbiome after FDR correction (all q > 0.05). Rainfall positively predicted the presence of an unknown family of Bacteroidetes (Χ^2^ = 5.902, p = 0.015, q = 0.014) in models that included hormones. Phytoprogestin period predicted the absence of the Coriobacteriaceae family of Actinobacteria (Χ^2^ = 4.588, p = 0.032, q = 0.028) and an unknown family within Proteobacteria (Χ^2^ = 8.349, p = 0.004, q = 0.003), and positively predicted the presence of the Bacteroidaceae family of Bacteroidetes (Χ^2^ = 4.852, p = 0.028, q = 0.024).

## Discussion

This study examined how gut microbiome composition varied across reproductive stages in Phayre’s leaf monkeys and whether this variation was correlated with fecal hormone concentrations. As hypothesized, we found differences in the gut microbiome during different stages of reproduction. These findings are consistent with a number of recent studies that have identified a shift in the gut microbiome related to changes in female reproductive state^2,4–9^. However, we also determined that, as hypothesized, hormone fluctuations appear to be driving these patterns. In particular we found that fP concentrations in Phayre’s leaf monkeys predicted gut microbiome diversity and community composition, and that coarse categories of reproductive state did not capture differences once hormones had been taken into account. Although reproductive hormones have been hypothesized as the main drivers of these changes, only one other study has identified a correlation between reproductive hormones and measures of microbiome diversity and composition^5^.

The direction and magnitude of change that we observed in the microbiome in response to pregnancy is consistent with a subset of what is reported in the literature. In models without hormones, we found that alpha diversity decreased during pregnancy, as has been reported in other vertebrates^3,4^, including humans^8^. However, these results contradict what has been found in other studies of humans, where alpha diversity is higher during pregnancy^49^ or shows no change^6,13^. While the underlying differences in physiological responses to pregnancy among host species could be causing these discrepancies, sampling differences may also contribute. Crusell *et al*. (2018) compared a single time point during pregnancy with postpartum samples, and both DiGiulio *et al*. (2015) and Jost *et al*. (2014) compared changes in alpha diversity longitudinally, but again only during pregnancy and postpartum. In fact, few studies sample females multiple times across all reproductive stages, including pre-conception, during gestation, and postpartum. We suggest that using fine scale longitudinal sampling prior to, during, and after pregnancy may be necessary to accurately describe microbiome fluctuations.

Furthermore, despite detecting an overall effect of reproductive stage on the gut microbiome, our results suggest that differences in diversity and composition across reproductive state may be driven by fP concentrations, similar to what was reported in a study of black rhinos (*Diceros bicornis michaeli*)^5^. fP was strongly associated with both diversity indices and gut microbiome composition, and fE exhibited a weak association with beta diversity. While there is some evidence that circulating estrogen levels influence bacterial quorum sensing and growth rates^16,17^, interactions between progesterone and the gut microbiome have rarely been reported. One single-species cell culture study suggested that higher progesterone concentrations increase the growth rate of *Bacteroides*^50^. However, with our small sample size, we were unable to detect the effects of reproductive state or reproductive hormone levels on the abundance of any individual bacterial taxa. Additionally, the number of poorly characterized taxa that could not be identified more specifically than class or family limited our ability to make functional inferences based on 16S rRNA sequences, as is often the case for wild study systems. Future studies should therefore incorporate a larger sample size, as well as metagenomic or metabolomic data, to address questions about the functional significance of changes in the gut microbiome associated with reproductive state and reproductive hormones. While our study focused on progesterone and estrogen, there are additional hormonal, metabolic, and immunological changes during pregnancy and lactation which may interact with the gut microbiome^15,51–53^. For instance, the changes in immune tolerance during pregnancy could result in increased abundances of potentially pathogenic bacteria within the gut microbiome. If possible, these related physiological changes should be considered in concert with hormones in future studies of the gut microbiome across reproductive state.

Although phytoprogestins did not alter the influence of fP on gut microbial composition, they appeared to have an independent effect on some aspects of the gut microbiome of Phayre’s leaf monkeys. Specifically, phytoprogestin consumption was associated with changes in community composition as measured with both unweighted and weighted UniFrac distances, an absence of taxa within Proteobacteria and Actinobacteria, as well as the presence of taxa within Bacteroidetes. However, the direction of the effect is somewhat surprising, as other studies have shown an increase in the relative abundance of Proteobacteria and Actinobacteria during pregnancy^7–9^. Nevertheless, the finding that fP concentrations influenced gut microbial composition regardless of phytoprogestin consumption lends strong support to the hypothesis that endogenous progesterone metabolites influence gut microbial communities. The additional independent effect of phytoprogestins on gut microbial composition may suggest that unknown plant compounds or hormone metabolites (not detected by our progesterone antibody) are also important. In particular, it is possible that metabolites of the same class of hormones (e.g., estrogens, androgens, or progestagens) have independent effects on gut microbial communities in addition to potential cross-talk between endogenous progesterone, phytoprogestin metabolites, and the gut microbiome. This possibility underscores the importance of unpacking hormone-microbiome relationships, both via experimental and correlational studies.

Consistent with previous studies, environmental factors associated with seasonality influenced gut microbial diversity and composition as well^12,54–61^. Rainfall predicted community composition and was positively associated with both Firmicutes and Bacteroidetes. Although the positive correlation between Firmicutes and rainfall is similar to results from wild baboons (*Papio cynocephalus*)^11^, Firmicutes were less abundant in the wet season in wild Verreaux’s sifakas (*Propithecus verreauxi*)^12^, and only some taxa within Firmicutes were more abundant during the wet season in wild white-faced capuchins (*Cebus capucinus*)^56^. Thus, while the relationship between the gut microbiome and aspects of seasonality such as rainfall and food availability are commonly found across studies, the specific taxa influenced by seasonality vary across primates. This is perhaps unsurprising. Given the strong influences of phylogeny, geography, and dietary niche on primate gut microbiomes^62–68^, the species or strains of microbes involved in dynamic shifts are likely to differ across ecoevolutionary contexts. In addition, fine scale dietary data that elucidate how nutrient consumption is changing during the wet season will allow us to better understand the interaction between the purported increase in food availability during the wet season, the resulting dietary changes, and the gut microbiome, as has been investigated in a limited number of studies^69,70^. Therefore, future research into the adaptive value of gut microbiome plasticity should investigate how different taxa might provide similar functional benefits to help individuals navigate the unique physiological challenges associated with energetic shortfalls or reproduction. How these microbial responses are mediated by changes in reproductive hormones should also be considered.

While data from the current study suggest that fP is strongly associated with shifts in the gut microbiome during pregnancy and lactation, we are constrained in our ability to determine how these changes influence host physiology or metabolism. First, a limited sample size prohibited our ability to identify the precise taxa that drove larger changes in diversity and composition. Additionally, using fecal samples alone does not capture the entire gut bacterial community that influences the nutrients available to the host, particularly in colobines. Phayre’s leaf monkeys, like other colobines, have sacculated forestomachs where much of their microbial fermentation occurs^23,71,72^. The microbial community in the foregut is distinct from that of the hindgut in colobines^73^. While the fecal microbiome is indicative of large-scale shifts in the overall gut microbiome^74^, the methods of the current study may prevent us from detecting how the gut microbiome as a whole is responding to changes in reproductive hormone concentrations and how those changes influence host metabolism. Finally, while the magnitude of the effects we report here are rather small, microbial shifts are but one mechanism that could be acting in concert with other behavioral and physiological strategies, such as allocare or dietary shifts, that influence female energetic strategies throughout reproduction. Additional data on host diet, energy balance, behavior, reproductive fitness, and gut microbial function will provide much needed context to determine the importance of these potentially hormone-mediated shifts in the gut microbiome during reproduction.

## Supplementary information

Supplementary Information.

Supplementary Information 2.

## Data Availability

Raw DNA sequences can be accessed through the Sequence Read Archive under BioProject ID PRJNA599430. Rainfall, reproductive state, and hormone data for the analysis are available at https://datadryad.org/stash/share/8r8NyiTlOn94oNV4kIcwOl_WFd6i2NmyDwUz1_AbjDk. Code for all analyses can be found at https://github.com/emallott/leafmonkeymicrobes.
