# Supplementary material for: Reproductive hormones mediate changes in the gut microbiome during pregnancy and lactation in Phayre’s leaf monkeys
Source: Sci Rep. 2020 Jun 19;10:9961. doi: 10.1038/s41598-020-66865-2 (PMC7305161; doi:10.1038/s41598-020-66865-2)
Supplement: Supplementary file 2 — Supplementary Information 2. [file 41598_2020_66865_MOESM2_ESM.docx]

**Title:** Reproductive hormones mediate changes in the gut microbiome during pregnancy and lactation in Phayre’s leaf monkeys

**Authors:** Elizabeth K Mallott, Carola Borries, Andreas Koenig, Katherine R Amato, Amy Lu

**Supplementary methods: Sequence processing details**

Using the QIIME2 (v2019.4) “dada2 denoise-paired” command: 1) 20 base pairs were trimmed from the 5’ end of both the forward and reverse reads to remove primers; 2) forward and reverse sequences were truncated at 290 base pairs and any sequences shorter than 290 base pairs were removed from the dataset; and 3) the expected error rate of 5 was used for forward and reverse reads.
